# WADDINGTON’S LANDSCAPE OF CELL AGING

**DOI:** 10.1093/geroni/igz038.755

**Published:** 2019-11-08

**Authors:** Nan Hao, Yang Li, Yanfei Jiang, Julie Paxman, Richard O’Laughlin, Lorraine Pillus, Lev Tsimring, Jeff Hasty

**Affiliations:** UC San Diego, La Jolla, California, United States

## Abstract

Cellular aging is a complex process that involves many interwoven molecular processes. Studies in model organisms have identified many individual genes and factors that have profound effects on lifespan. However, how these genes and factors interact and function collectively to drive the aging process remains unclear. We investigated single-cell aging dynamics throughout the replicative lifespans of S. cerevisiae, and found that isogenic cells diverge towards two aging paths, with distinct phenotypic changes and death forms. We further identified specific molecular pathways driving each aging fate and revealed that these pathways interact and operate dynamically to enable an early-life switch that governs the aging fate decision and the progression towards death. Our work uncovers the interconnected molecular pathways that drives the aging process and opens up the possibility of designing interventions that simultaneously target multiple network nodes, instead of single genes, to more effectively extend the healthspan.

